# Comparative Immunohistochemical Study of Multicystic Dysplastic Kidneys With and Without Obstruction

**DOI:** 10.3109/15513815.2011.572960

**Published:** 2011-06-20

**Authors:** Claudia P Rojas, Arnel K Urbiztondo, Jocelyn H Bruce, Maria M Rodriguez

**Affiliations:** Department of Pathology, University of Miami, Holtz's Children's Hospital, Miami, Florida, USA

**Keywords:** autopsies, developmental pathology, immunohistochemistry, nonobstructive multicystic dysplastic kidney, obstructive multicystic dysplastic kidney, transcription growth factor

## Abstract

Etiology of multicystic dysplastic kidney (MCDK) remains unknown. Not all cases are associated with obstruction. We compared by immunohistochemistry 17 cases of MCDK (10 cases with and seven without obstruction) to 17 controls and 20 fetal kidneys. TGF-β was negative in obstructive MCDKs and positive in nonobstructive MCDK. IGF2 was overexpressed in obstructive and underex-pressed in nonobstructive MCDKs. PAX2, BCL-2, and β-catenin were expressed equally in obstructive and nonobstructive dysplasia. TGF-β and IGF2 work by different mechanisms in obstructive and nonobstructive MCDKs, but there are no differences among PAX 2, BCL-2, and β-catenin in obstructive versus nonobstructive dysplasia.

## INTRODUCTION

The etiology of multicystic dysplastic kidney (MCDK) is not well understood. While there are many kidneys with evidence of obstruction at the level of the renal pelvis, ureters, bladder, or urethra, there is another subset of patients with MCDKs in which there is no evidence of obstruction. These patients may have an isolated renal defect or other major malformations such as in Meckel-Gruber syndrome, polysplenia, short-rib syndrome, congenital heart disease, trisomies, etc.

Multicystic dysplastic kidney occurs in 1 out of 4300 live births [1]. Multicystic dysplasia involves the interaction betweeen the metanephric blastema and the ureteric bud. Multicystic dysplastic kidney is generally associated with an imbalance in the normal mesenchymal to epithelial transformation resulting in excessive fibrous tissue surrounding cystically dilated tubules and a decreased number of glomeruli. Transcription growth factor-beta (TGF-β) is important in the mesenchymal-to-epithelial transformation process involving the metanephric blastema and the ureteric bud [2]. Alterations in the regulatory genes such as paired box gene 2 (PAX2) [3] and the proto-oncogene B-cell lymphoma-2 (BCL-2) [4] are associated with the formation of renal cysts. PAX2 is a transcription factor that is active in nephrogenesis, including condensing metanephric mesenchyme and in early epithelial structures derived from mes-enchyme. PAX 2 is downregulated as the tubular epithelium matures. BCL-2 represses cell death by apoptosis; the later has been proven to be related to the development of polycystic kidneys, hydronephrosis, glomerular conditions, and ischemic renal atrophy. Insulin growth factor 2 (IGF 2) is expressed in fetal kidney and has been demonstrated in cystic epithelium of MCDK [5], but to the best of our knowledge, there are no studies comparing the expression of these immunohistochemical stains in obstructed versus nonobstructed MCDKs.

This article characterizes the immunohistochemical profiles of TGF- β, PAX2, BCL-2, β-catenin, and IGF2 in MCDK with and without obstruction, compared to normal kidneys (using tissue from human autopsies) and fetal-embryonal kidneys from the surgical pathology files.

## METHOD

The autopsy files at our institution were searched for patients with MCDK and age-matched controls. The Institutional Review Board (IRB) approved the protocol prior to collecting the cases. The fetuses and embryos were selected from the surgical pathology files. The lower limit of gestational age for the embryos was 5 weeks and the upper limit for the fetuses was 19 weeks. The age range for autopsies was from 20 to 41 weeks of gestation. The hematoxylin-and-eosin (H&E)-stained slides were reviewed and blocks containing kidneys were identified.

Paraffin sections were cut from the selected blocks and stained by immunohisto-chemistry for TGF-β, PAX2, BCL-2, β-catenin, and IGF-2. We used the mouse monoclonal TGF-β (Abeam, Cambridge, MA) diluted 1:1000, anti-PAX2 polyclonal antibody (Zymed Laboratories Inc., South San Francisco, CA) at a dilution of 1:100. Anti-human BCL-2 oncoprotein antibody (Dako, Carpinteria, CA) was diluted 1:40. Anti- β-catenin (Upstate Biotechnology, Lake Placid, NY) was diluted 1:100; rabbit polyclonal antibody to insulin growth factor (Abeam, Cambridge, MA) was diluted at 1:100.

The pattern of staining was interpreted and recorded as negative or when positive as nuclear, cytoplasmic, and/or membranous. Cases with MCDK (obstructive and nonobstructive) were mixed and examined blindly by two pathologists. The results were recorded and further separated into two groups. Likewise, the age-matched controls and embryo-fetal kidneys were also reviewed by two pathologists and the pattern of staining was recorded. A total of 17 cases with MCDK, 17 controls and 20 embryos/fetuses were studied.

## RESULTS

**FIGURE 1:**
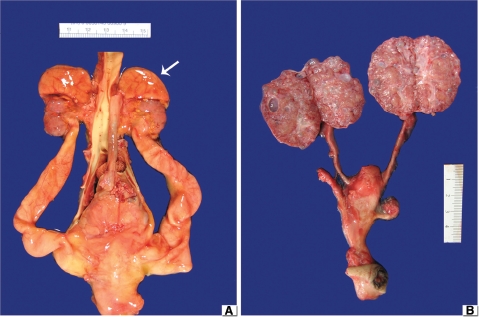
(**A**) Obstructive MCDK. Notice the hypoplastic kidneys with the adrenal glands still at the upper poles (arrow). The aorta is centrally located and there are bilateral hydroureters. (**B**) Nonobstructive MCDK. The ureters are unremarkable and portions of the two umbilical arteries run parallel to the urinary bladder. The kidneys are bi-valved to demonstrate numerous small cysts scattered through the cortex and medulla. 101 × 67mm.

Of the 17 cases with MCDK's, 10 were associated with obstruction and 7 did not show evidence of obstruction. The clinical information of cases with obstruction are shown in Table 1, while Table 2 details the patients without genito-urinary obstruction. Macroscopic examples of obstructive and nonobstructive MCDK are shown in Figure 1.

**TABLE 1:** Patients with Multicystic Dysplastic Kidneys with Associated Obstruction. Immunohistochemistry and Clinical Information

Case#	Sex	GA Weeks	Age	TGF-β	PAX2	BCL2	β Catenin	IGF2	Clinical Information
1	M	37	1 day	Negative	Nuclear DTE	Neg	Memb DTE	Memb DTE	Prune belly syndrome: atretic distal urethra, dilated bladder, tortuous hydroureters, bilateral hypoplastic MCDK.	
2	M	36	1 day	Negative	Nuclear DTE	Memb DTE	Memb DTE	Neg	Prune belly syndrome: posterior urethral valves, dilated bladder, bilateral hydroureters, and MCDK.	
3	M	37	1 day	Negative	Nuclear DTE	Memb DTE	Memb DTE	Memb DTE	Prune belly syndrome: atretic urethra,markedly dilated and hypertrophic bladder, bilateral hydroureters, and hypoplastic MCDKs.	
4	M	36	24 days	Negative	Nuclear DTE	Neg	Memb DTE	Memb DTE	Prune belly syndrome: atretic distal urethra, dilated and hypertrophic bladder, tortuous hydroureters, hypoplastic MCDKs.	
5	M	38	3 days	Negative	Nuclear DTE	Memb DTE	Memb DTE	Memb DTE	Right renal agenesis, left MCDK, hypoplastic urinary bladder with a recto-vesical fistula (meconium coming thru urethra), ambiguous external genitalia.	
6	M	37	1 hour	Negative	Nuclear DTE	Memb DTE	Memb DTE	Memb DTE	Right renal agenesis, left MCDK, hypoplastic urinary bladder, single umbilical artery.
7	M	32	1 day	Negative	Nuclear DTE	Memb DTE	Memb DTE	Memb DTE	Prune belly syndrome: posterior urethral valves, dilated bladder, bilateral hydroureters, MCDKs.	
8	M	20	0	Negative	Nuclear DTE	Memb DTE	Memb DTE	Memb DTE	VACTER-L. Vertebral anomalies, esophageal atresia, tracheo-esophageal fistula, bilateral absence of fibula and patella, congenital heart disease, posterior urethral valve, bilateral MCDKs.	
9	M	39	48 days	Negative	Nuclear DTE	Memb DTE	Memb DTE	Memb DTE	Bilateral hypoplastic MCDKs. Urethra opening into the scrotum.	
10	F	17	0	Negative	Nuclear DTE	Memb DTE	Memb DTE	Memb DTE	Esophageal atresia, low-set ears,micrognathia, congenital heart disease, right MCDK with hydronephrosis, normal left kidney, imperforate anus, clitoral hypertrophy, karyotype 47,XXX.

Abbreviations: GA = gestational age; +positive; DTE = dysplastic tubular epithelium; Neg = negative;Memb = membranous.

**TABLE 2:** Patients with Multicystic Dysplastic Kidneys without Associated Obstruction. Immunohistochemistry and Clinical Information

Case #	Sex	GA Weeks	Age	TGF-β	PAX2	BCL2	B Catenin	IGF2	Clinical Information
1	M	40	1 hour	+ IT	Nuclear DTE	Cytop.	Memb.	Weakmemb & cytop	Meckel Gruber syndrome.MCDKs, congenital hepatic fibrosis, polydactyly.
2	M	17	0	+ IT	Nuclear DTE	Cytop.	Memb.	Weakmemb & cytop	Trisomy 18. Congenital heart disease, bilateral MCDKs.
3	M	38	21 days	+ IT	Nuclear DTE	Cytop.	Memb.	Weakmemb & cytop	Ivemark syndrome (polysplenia, congenital heart disease, MCDKs, congenital hepatic fibrosis, and pancreatic cystic dysplasia).
4	M	40	6 days	Neg	Nuclear DTE	Cytop.	Memb.	Weakmemb & cytop	MCDKs, congenital heart disease, congenital diaphragmatic hernia, omphalocele.
5	M	32	1 day	+ IT	Nuclear DTE	Cytop.	Memb.	Weakmemb & cytop	Polysplenia, congenital heart disease, left MCDK, normal right kidney.
6	F	32	1 day	Neg.	Nuclear DTE	Cytop.	Memb.	Weakmemb & cytop	Twin B, oligohydramnios sequence, nonsyndromic, bilateral MCDKs (normal twin A).
7	M	28	1 day	+ IT	Nuclear DTE	Cytop.	Memb.	Weakmemb & cytop	Meckel Gruber syndrome.MCDKs, congenital hepatic fibrosis, polydactyly.

Abbreviations: GA = gestational age; IT = interstitium; DTE = dysplastic tubular epithelium;Memb = membranous; cytop = cytoplasmic.

**FIGURE 2:**
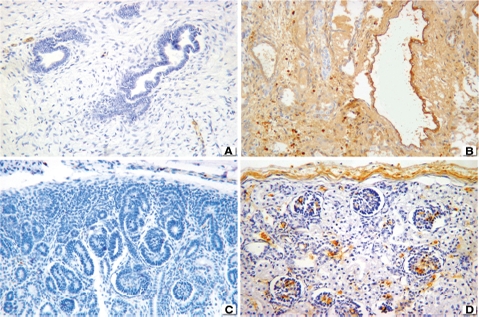
TGF-β staining. (**A**) Negative in obstructive MCDK. (**B**) Positive staining in interstitium and tubular epithelium of nonobstructive (syndromic) MCDK. (**C**) Negative staining in tubules including nephrogenic zone of fetus. (**D**) Focal staining in glomeruli of controls. 76 × 57 mm.

The immunohistochemical profile is described as follows: TGF-β was negative in 10 out of 10 cases with MCDK associated with obstruction (Figure 2A), while it was positive in the renal interstitium of five patients with syndromic nonobstructive MCDK (Figure 2B). TGF-β was negative in the two patients with nonsyndromic, nonobstructive MCDK. All fetuses and embryos were negative in the interstitium and in tubules and nephrogenic zone (Figure 2C). Regarding controls, TGF-β was uniformly negative in the interstitium, but it was focally positive in glomeruli (Figure 2D).

**FIGURE 3:**
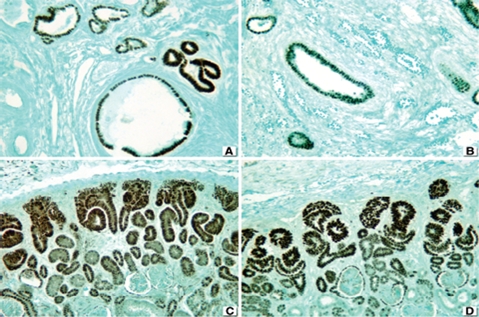
PAX2. (**A**) Positive staining in dysplastic tubular epithelium and proximal tubules in obstructive MCDK. (**B**) Nonobstructive MCDK, positive staining in dysplastic tubular epithelium and proximal tubules. (**C**) Strong staining in fetal kidney. (**D**) Over-expression of PAX2 in nephrogenic zone of immature kidney (less than 36 weeks). 76 × 57 mm.

PAX2 depicted a nuclear staining in the dysplastic tubular epithelium and proximal tubules of all obstructive (Figure 3A) and nonobstructive MCDK's (Figure 3B.) Likewise, the embryos and fetuses were all positive in the nephrogenic zone (Figure 3C). In controls with a gestational age of less than 36 weeks, there was active nephrogenesis and the nephrogenic zone and medullary tubules were positive for PAX2; however, the more mature tubules were either weakly positive or negative (Figure 3D).

**FIGURE 4:**
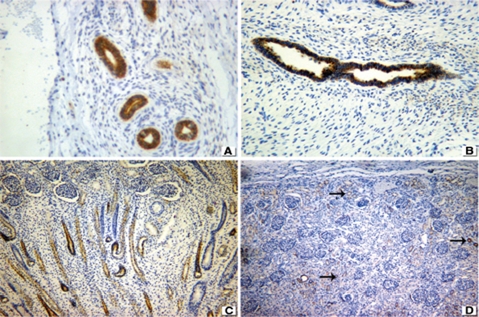
BCL2 expression. (**A**) Positive cytoplasmic staining in dysplastic tubular epithelium of obstructive MCDK. (**B**) Similar positivity in non-obstructive MCDK. (**C**) Positive in nephro-genic zone of fetal kidney. (**D**) Minimal expression in the tubules of normal control (arrows). 76 × 57 mm.

BCL-2 demonstrated a membranous pattern in dysplastic tubular epithelium in 8 out of 10 cases with obstructive MCDK (Figure 4A). All seven nonobstructive MCDK were positive for BCL-2 in dysplastic tubular epithelium, while the mesenchyme was negative in all dysplastic kidneys (Figure 4B). All preterm controls with evidence of nephrogenesis depicted a membranous pattern of staining. BCL-2 was positive in the nephrogenic zone of fetal and embryonal kidneys (Figure 4C); it was negative in the interstitium of normal kidneys but stained positive in some tubules (Figure 4D - see arrows).

**FIGURE 5:**
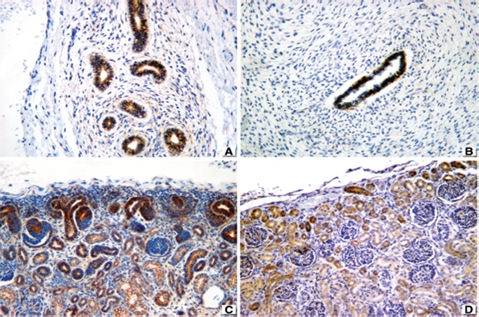
Beta-catenin panel. (**A**) Membranous staining in dysplastic tubular epithelium of a case with obstructive MCDK. (**B**) Same pattern in a patient with nonobstructive MCDK. (**C**) Overexpres-sion in nephrogenic zone of fetal kidney. (**D**) Positive reaction in normal tubular epithelium of control. 76 × 57 mm.

β-catenin showed a membranous staining of dysplastic tubular epithelium in 10 out of 10 cases with obstructive (Figure 5A) and in all seven patients with nonobstructive MCDKs (Figure 5B). The staining was even stronger in the nephrogenic zone of fetal and embryonal kidneys (Figure 5C) and controls (Figure 5D).

**FIGURE 6:**
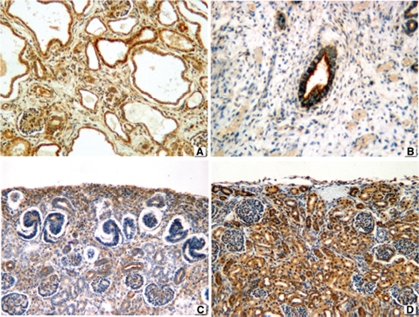
IGF2 expression in (**A**) obstructive MCDK depicts strongly positive membranous and cytoplasmic staining. (**B**) Weaker membranous expression in non-obstructive MCDK. (**C**) Positive staining in nephrogenic zone of preterm kidney. (**D**) Positive normal control. 76 × 57 mm.

IGF-2 exhibited a strong membranous and cytoplasmic pattern in the dysplastic tubular epithelium of 9 out of 10 patients with obstructive MCDK (Figure 6A) and a weaker, albeit positive, staining in all seven cases with nonobstructive MCDKs (Figure 6B). It was positive in the nephrogenic zone of all fetal and embryonal kidneys (Figure 6C). Regarding normal controls, IGF-2 was positive (membranous and cytoplasmic) in the cortical tubular epithelium in 15 out of 17 patients (Figure 6D).

## DISCUSSION

TGF-β is a secreted growth factor that is part of a family of proteins known as a transforming growth factor beta superfamily, which includes inhibins, activin, anti-mullerian hormone, bone morphogenetic protein, decapentaplegic, and Vg-1. TGF-β is involved in numerous cellular functions including proliferation and differentiation. It is also implicated in epithelial-to-mesenchymal transformation that is an important component of organogenesis. *In-vitro* studies showed TGF-β to cause mammalian epithelia and endothelia to develop mesenchymal characteristics. TGF-β is expressed during normal rodent nephrogenesis and is upregulated in the sheep model of fetal kidney obstruction [6]. These authors found immunoreactive TGF-β in the urine after experimental obstruction of ovine fetal lower urinary tract. In our study, there was no immunoreactivity for TGF-β in MCDKs associated with obstruction, while on the other hand, we found all cases of syndromic MCDK without evidence of obstruction to be immunoreactive for TGF-β (see Tables 1 and 2 for clinical information).

It has been proven that *stumpy* mutant mice have a defect in ciliogenesis resulting in hydrocephalus and cystic renal disease resembling the human Meckel Gruber syndrome [7]. These authors deleted a genomic region of the brain by crossing floxed (fl) mice to *nestin*-cre deleter transgenic animals and defined the deleted alleles in ho-mozygous and heterozygous fl mice. They found that TGFbl mRNA expression was increased to 1.8 to 3.0 fold in fl/fl vs. +/fl mice. We hypothesize that our results of positive TGF staining in patients with Meckel Gruber and polysplenia could be explained by a primary defect in renal cilia as the cause of multicystic renal dysplasia, while the patients with a primary genito-urinary obstruction develop the cysts by a different mechanism such as increased hydrostatic pressure in the tubules.

Paired box (PAX) genes are a family of tissue-specific transcription factors containing a paired domain. Paired box genes were initially described in the Drosophila. They are expressed during embryogenesis and are important in early development for the specification of tissues. There are a total of nine PAX genes in humans. Two of these PAX genes are involved in nephrogenesis—PAX2 and PAX8. However, only PAX2 is implicated in renal disease. Transgenic overexpression of PAX2 leads to epithelial hyper-proliferation and cyst formation. On the other extreme, absence of a single PAX2 allele will cause renal hypoplasia. Reduced PAX2 protein expression inhibits mesenchymal to epithelial transition which is important in nephrogenesis. PAX2 has been localized in the various parts of the developing kidney including the mesenchymal condensate, S-shaped bodies, and tips of ureteric buds. In the dysplastic kidney, PAX2 has been found in the dysplastic tubular epithelia. It is, however, absent in the collaretes [3]. In our study, we found that PAX2, consistent with being a transcriptional factor, is present within the nuclei of dysplastic tubular epithelium in MCDK cases and the nuclei of nondysplastic tubular epithelium of preterm controls. It is also present in the immature glomeruli within the nephrogenic zones of controls and fetal-embryonic cases. However as the gestational age of MCDK cases and controls increased, the intensity of immunostaining diminished. Immunostaining for PAX2 was negative in the mature glomeruli.

BCL-2 is a proto-oncogene originally associated with the t(12:18) chromosomal translocation, which is the cytogenetic hallmark of follicular lymphoma [4]. As a member of a family of proteins believed to regulate programmed cell death, subsequent studies have shown the ability of BCL2 to inhibit apoptosis. It is expressed in a variety of fetal tissue including the developing kidney. Studies with BCL-2 showed intense staining of the glomerulogenic zone subjacent to the renal capsule and the absence of staining in a normal kidney with the exception of positivity in the parietal epithelium of the Bowman's capsule [8]. Our findings support this as we observed strong expression of BCL-2 in the fetal-embryonal kidney with subcapsular staining that formed a cap around the S-shaped bodies. As the gestational age increased, the intensity of staining diminished due to the downregulation of the gene expression as shown by weaker expression in the glomerular epithelium and tubules.

Previous murine studies have documented the importance of BCL-2 in normal nephrogenic development [9]. Genetically engineered mice with blocked expression of BCL-2 led to excessive apoptosis of the metanephric blastema causing polycys-tic kidneys and severe renal failure. We expected therefore that BCL-2 should be markedly decreased or even absent in the dysplastic parenchyma and tubules of multicystic renal dysplasia. In our study, however, BCL-2 was present and stained the cytoplasm of the dysplastic tubules of 8 out of 10 cases of obstructive and all nonobstructive multicystic dysplastic kidneys. We are attributing this discrepancy to the development of newer and more sensitive antibodies currently in use that can now detect the protein in question. Alternatively, our findings suggest that BCL-2 may contribute to the development of cysts through mechanisms other than the apoptosis pathway.

β-catenin is a bi-functional glycoprotein involved in cell-cell adhesion and in gene transcription that forms an integral part of the Wnt signaling pathway which has been found to regulate nephron induction during mouse kidney development. Hu and Rosenblum [10] observed the increased β-catenin expression in the medulla of dys-plastic kidney tissue from genetically altered mice that expressed an active form of activin-like kinase (Alk)3, a bone morphogenetic protein (bmp) cell surface receptor. This bmp receptor is expressed in both the metanephric blastema and ureteric bud during early renal development. The active form of Alk3, Alk3^QD^, when overexpressed, led to the formation of medullary cystic dysplasia characterized by a kidney with a decreased number of medullary tubules, loss of epithelial differentiation markers, and cystic malformation of epithelial tubules. On the other hand, Bridgewater et al. [11] used other genetically altered mice with β-catenin deficiency targeted at the ureteric bud lineage which developed bilateral renal aplasia or renal dysplasia.

Insulin growth factor has been proven to be overexpressed in multicystic renal dysplasia [5]. However, a search in the English medical literature did not show any article addressing the pattern of expression in the two major types of MCDKs. We found greater overexpression of IGF2 in obstructive MCDK compared to syndromic nonob-structive MCDK. The staining was also found in the fetal nephrogenic zone. Interestingly, our controls stained the cortical tubular epithelium with a membranous and cytoplasmic pattern.

We postulate that the etiology of cyst formation in nonobstructive syndromic MCDK may be related to an abnormality of the cilia. Patients with Meckel Gruber syndrome have, among other features, a co-existence of MCDK with congenital hepatic fi-brosis and sometimes pancreatic cysts. A group from Germany published the study of a homozygous *Nphp*^3^ -deficient animal model to determine the role of nephrocystin-3 during early development and left-right body asymmetry, heterotaxia, congenital heart disease, and embryonic lethality [12]. These mice had longer cilia than normal controls by immunofluorescence. The authors demonstrated in patients and mice that NPHP3/Nphp3 mutations produce several clinical manifestations such as *situs inversus*, polydactyly, central nervous system anomalies, congenital heart disease, preau-ricular fistulas, and several renal malformations including MCDK. Further genetic studies of larger series of syndromic vs. nonsyndromic patients with renal dysplasia are needed to confirm the role of TGF-β in syndromic nonobstructive MCDK.
